# Human periodontal ligament stem cell sheets activated by graphene oxide quantum dots repair periodontal bone defects by promoting mitochondrial dynamics dependent osteogenic differentiation

**DOI:** 10.1186/s12951-024-02422-7

**Published:** 2024-03-27

**Authors:** Na An, Xiaoyuan Yan, Qiujing Qiu, Zeying Zhang, Xiyue Zhang, Bowen Zheng, Zhenjin Zhao, Jiajie Guo, Yi Liu

**Affiliations:** 1 Department of Orthodontics, School and Hospital of Stomatology, Liaoning Provincial Key Laboratory of Oral Diseases, China Medical University, Shenyang, 110002 China; 2 Department of Endodontics, School and Hospital of Stomatology, Liaoning Provincial Key Laboratory of Oral Diseases, China Medical University, Shenyang, 110002 China

**Keywords:** Graphene oxide quantum dots, Periodontal ligament stem cells, Periodontal bone defect, Bone regeneration, Mitochondrial dynamics

## Abstract

**Background:**

Bone defects in the maxillofacial region restrict the integrity of dental function, posing challenges in clinical treatment. Bone tissue engineering (BTE) with stem cell implants is an effective method. Nanobiomaterials can effectively enhance the resistance of implanted stem cells to the harsh microenvironment of bone defect areas by promoting cell differentiation. Graphene oxide quantum dots (GOQDs) are zero-dimensional nanoscale derivatives of graphene oxide with excellent biological activity. In the present study, we aimed to explore the effects of GOQDs prepared by two methods (Y-GOQDs and B-GOQDs) on the osteogenic differentiation of human periodontal ligament stem cells (hPDLSCs), as well as the effect of gelatin methacryloyl (GelMA)-encapsulated GOQD-induced hPDLSC sheets on the repair of mandibular periodontal defects in rats. We also explored the molecular biological mechanism through which GOQD promotes bone differentiation.

**Results:**

There were significant differences in oxygen-containing functional groups, particle size and morphology between Y-GOQDs and B-GOQDs. Y-GOQDs promoted the osteogenic differentiation of hPDLSCs more effectively than did B-GOQDs. In addition, GelMA hydrogel-encapsulated Y-GOQD-induced hPDLSC cell sheet fragments not only exhibited good growth and osteogenic differentiation in vitro but also promoted the repair of mandibular periodontal bone defects in vivo. Furthermore, the greater effectiveness of Y-GOQDs than B-GOQDs in promoting osteogenic differentiation is due to the regulation of hPDLSC mitochondrial dynamics, namely, the promotion of fusion and inhibition of fission.

**Conclusions:**

Overall, Y-GOQDs are more effective than B-GOQDs at promoting the osteogenic differentiation of hPDLSCs by regulating mitochondrial dynamics, which ultimately contributes to bone regeneration via the aid of the GelMA hydrogels in vivo.

**Graphical Abstract:**

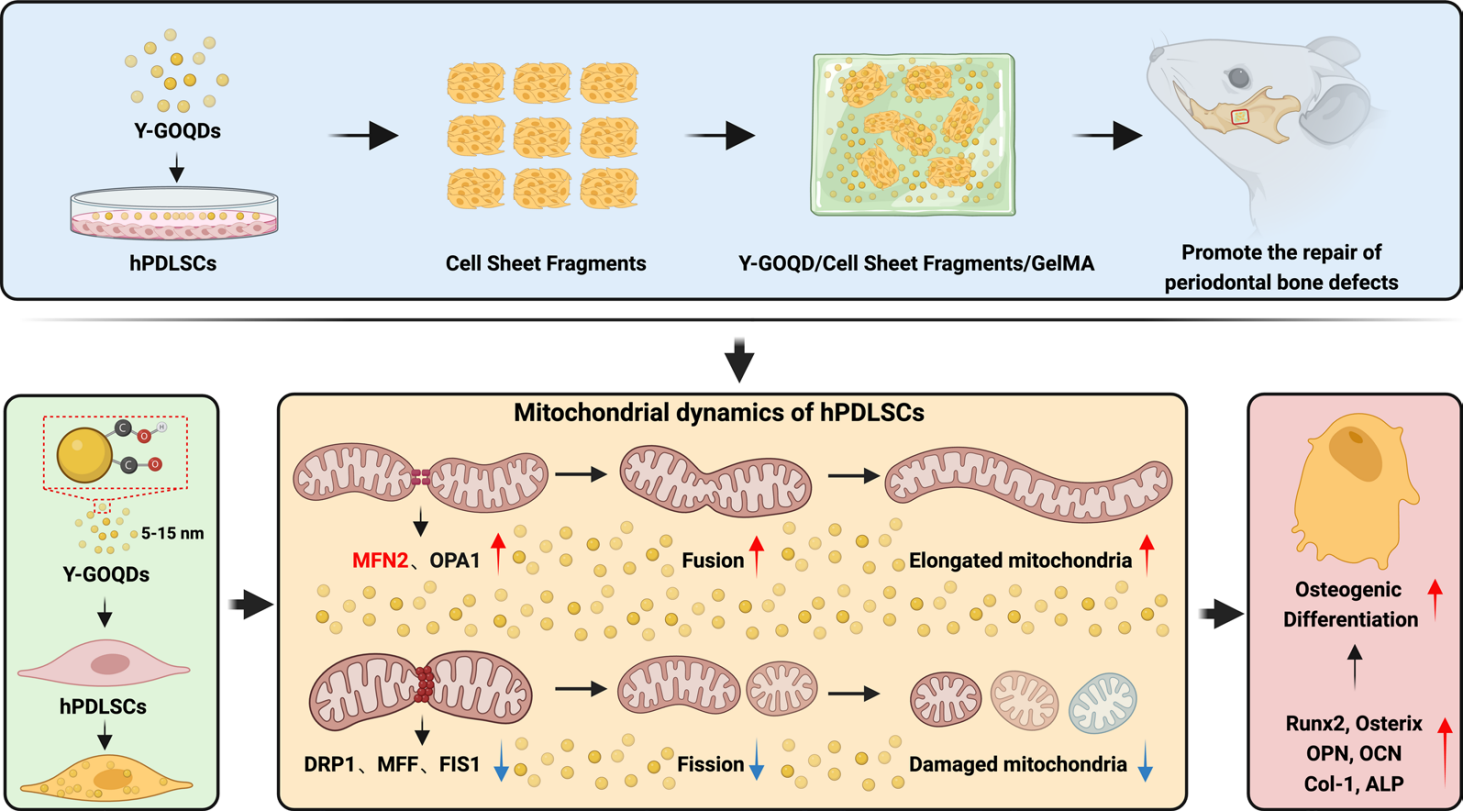

**Supplementary Information:**

The online version contains supplementary material available at 10.1186/s12951-024-02422-7.

## Background

Many diseases in the oral and maxillofacial regions, such as periodontal disease, periapical disease, bone dehiscence, bone fenestration, and jaw tumors, are accompanied by the destruction of periodontal and bone tissue. Such destruction often exceeds the body's ability to heal and repair, severely restricting oral function, and is currently a challenge in clinical treatment. The development of cell-based bone tissue engineering (BTE) has led to the development of new strategies for the regeneration of periodontal bone tissues. Through the action of bioactive factors, exogenous seed cells loaded with scaffold materials can effectively address the problems of inadequate bone-forming cells in lesion areas and repair periodontal bone defects [1–3].

Human periodontal ligament stem cells (hPDLSCs) are a type of mesenchymal stem cell (MSC). They are isolated and cultured from human periodontal ligaments. Under specific conditions, hPDLSCs can self-renew and differentiate into various types of tissues, such as periodontal connective tissue, dentin, alveolar bone, muscle, and nerves. hPDLSCs play crucial roles in maintaining and remodeling periodontal tissue, making them ideal "seed cells" for periodontal tissue repair and regeneration [4–6]. Combining expanded or induced hPDLSCs with biomaterials can effectively promote tissue repair and regeneration in periodontal defect areas [7]. However, allogeneic transplantation may cause immune responses or inflammation [8]. The microenvironment of long-term bone defect areas is also unfavorable for bone tissue growth. In immune, infectious, and low-nutrient environments, graft stem cells exhibit impaired bone-forming activity by producing excessive amounts of intracellular reactive oxygen species [9–11]. Therefore, bioactive factors that promote the survival rate and differentiation potency of stem cells are still a research hotspot in BTE.

Graphene oxide quantum dots (GOQDs) are new carbon-based nanoscale particles. They are derivatives of graphene oxide (GO) and are ultrasmall nanosheets of GO. The size of GOQDs is approximately 20 nm, which is much smaller than that of GO [12–14]. Approximately zero-dimensional GOQDs exhibit low cytotoxicity [15, 16]. GOQDs can quickly penetrate through the cell membrane without disrupting the lipid membrane structure. GOQDs contain abundant oxygenated functional groups, which facilitate their excellent dispersibility in water and high biological activity [17–19]. It has been shown that GOQDs can effectively promote the osteogenic differentiation of dental pulp stem cells, stem cells from human exfoliated deciduous teeth and MSCs in vitro [20–23]. The facilitation of osteogenic activity by GOQDs allows them to function as bioactive factors in BTE. However, different biological responses are induced by GOQDs in different cells, and the effective concentration of GOQDs can differ among cells. There is currently no research on whether GOQDs can promote the osteogenic differentiation of hPDLSCs. Most of the studies on GOQD-induced odontogenic stem cells have been conducted in vitro. However, further experimentation is needed to determine whether GOQD-treated stem cells can effectively repair bone defects in vivo. In addition, the proper method for transplanting GOQD-treated stem cells into surgical sites also needs exploration.

GOQDs are mainly prepared by two methods, namely, the “top-down” and “bottom-up” routes. “Top-down” methods usually utilize techniques such as electrochemical oxidation, laser ablation, and arc discharge to decompose larger carbon sources, including graphite, carbon nanotubes, and carbon black [24, 25]. The "bottom-up" methods involve combustion or solvothermal techniques for processing small molecule precursors such as citrate, carbohydrates, glucosamine, and ascorbic acid [13]. GOQDs obtained from different fabrication techniques may vary in particle size, edge structure, type of oxygen functional groups, and fluorescence performance, resulting in changes in their biological, optical, and electronic properties [13, 26]. However, for GOQDs prepared by different processes, few studies have compared differences in their physicochemical properties and osteogenic-promoting abilities.

To date, most studies of the effects of biomaterials on stem cells have focused on changes in the cell phenotype or the nuclear genome. GOQDs have been shown to promote the differentiation of odontogenic stem cells, and existing studies have largely attributed this effect to the activation of classic osteogenic signals, such as those in the Wnt pathway or autophagy pathway [20, 22, 23]. However, the mechanism involved in this process has largely not been elucidated. Mitochondria are the main energy supply centers of cells and are closely related to cellular behavior. They play an important regulatory role in the differentiation of MSCs [27, 28]. Mitochondria are highly dynamic organelles that undergo fusion and fission; these processes are collectively termed mitochondrial dynamics [29]. Fusion enables mtDNA, protein, or metabolite exchange between mitochondria. Fission separates damaged areas of dysfunctional mitochondria, leading to the removal of dysfunctional mitochondria through mitophagy [30]. In a recent study, mitochondrial fission and donut formation were shown to enhance mitochondrial secretion to promote osteogenesis in mature osteoblasts [31]. However, a previous study revealed that the upregulation of mitochondrial dynamics via both fusion and fission is responsible for the osteogenic differentiation of MSCs [32]. These findings suggested that changes in mitochondrial dynamics vary greatly during osteogenic differentiation in different cells. Therefore, exploring whether GOQDs can modulate mitochondrial dynamics to influence the osteogenic differentiation of hPDLSCs is worthwhile.

In this study, we used GOQDs prepared by two different methods. One type of GOQD was prepared using a “top-down” approach by decomposing graphite [33] and exhibited yellow‒brown fluorescence under ultraviolet (UV) light; these GOQDs are abbreviated as Y-GOQDs. The other type of GOQD was prepared using a “bottom-up” approach by pyrolyzing citric acid [34] and exhibited blue fluorescence under UV light; these GOQDs are abbreviated as B-GOQDs. We compared the physicochemical properties as well as the effects and mechanisms of the two types of GOQDs on the osteogenic differentiation of hPDLSCs. Y-GOQDs contained more oxygen functional groups than did B-GOQDs. Gelatin methacryloyl (GelMA)-encapsulated Y-GOQD-induced hPDLSC sheets effectively promoted the repair of mandibular periodontal defects in rats. Y-GOQDs enhanced the osteogenic differentiation of hPDLSCs by upregulating mitochondrial fusion and inhibiting mitochondrial fission. Compared with Y-GOQDs, B-GOQDs had a relatively weaker effect on promoting osteogenic differentiation and mitochondrial fusion.

## Materials and methods

### Cell isolation, culture, differentiation, and identification

hPDLSCs were isolated from the periodontal ligaments of premolars extracted from orthodontic patients (18–25 years old) under approved guidelines established by the Ethics Committee of the School of Stomatology, China Medical University, Shenyang, China (2020036). The periodontal ligament tissue from the middle section of the roots was mechanically minced and cultured in α-MEM (01-042-1ACS; Biolnd, Israel) supplemented with 10% fetal bovine serum (FBS; 164210; Procell, China) and 1% (v/v) penicillin/streptomycin (SV30010; HyClone, USA), with a medium change every 3 days. hPDLSCs at passage numbers between 3 and 7 were used for the experiments.

hPDLSCs (1.5 × 10^5^ per well) were seeded in 12-well plates and grown for 24 h. For osteogenic differentiation, the medium was replaced with low-glucose DMEM (01-051-1ACS, Biolnd) supplemented with 50 μg/mL ascorbic acid (A0278; Sigma, USA), 10 mM β-GP (G5422; Sigma) and 100 nM dexamethasone (D4902; Sigma) for the indicated lengths of time. For adipogenic differentiation, the medium was replaced with low-glucose DMEM supplemented with 0.01 mg/mL insulin (I8830; Solarbio, China), 0.2 mM indomethacin (I106885; Aladdin, China), 1 μM dexamethasone and 0.5 mM IBMX (I106812; Aladdin) for 21 days. The differentiation medium was changed every 3 days.

For flow cytometry-based identification of hPDLSCs, 1 × 10^6^ hPDLSCs were resuspended and washed with phosphate-buffered saline (PBS) 2–3 times. Then, 100 μL of cell suspension containing 3% FBS was incubated with 5 μL of PE-conjugated anti-human CD14 (301805; Biolegend, USA), PE-conjugated anti-human CD45 (368509; Biolegend), PE-conjugated anti-human CD29 (303003; Biolegend), PE-conjugated anti-human CD105 (800503; Biolegend), or PE-conjugated anti-human CD146 (361005; Biolegend) for 20 min in the dark. After the cells were washed twice with PBS, a flow cytometer (BD Biosciences, USA) was used to analyze the cells.

### GOQD characterization

GOQDs (XF074-1, XF042-1) were purchased from Nanjing XFNANO Materials Tech Co., Ltd. (Nanjing, China). The diameter of the GOQDs was measured using high-resolution transmission electron microscopy (HRTEM, JEM-2100F, JEOL, Japan). The particle size was statistically analyzed by Nano Measure software. The surface morphology of the GOQDs was obtained by using an atomic force microscope (JEM-2100F, AFM, Shimadzu, Japan), and the particle thickness was calculated using Gwyddion software. The oxygen-containing functional groups of the GOQDs were determined by using a Fourier transform infrared spectrometer (VATAR 360, Nicolet, USA). The X-ray photoelectron spectroscopy (XPS) results for the GOQDs were obtained by using a spectrometer (Escalab 250Xi, Thermo Fisher Scientific, USA), and the binding energy of the GOQDs was calibrated with O 1 s and C 1 s as the benchmark to further clarify the existence and content of various functional groups.

### Cell viability

hPDLSCs (5 × 10^3^ per well) were seeded in 12-well plates and grown for 24 h. The next day, the cells were incubated with GOQDs at a range of concentrations for 1, 3, or 5 days. Then, the medium was replaced with growth medium containing 10% Cell Counting Kit-8 (CCK-8; K1018; APExBIO, USA) solution. After a 1 h of incubation at 37 °C, the absorbance at 450 nm was measured using a microplate reader (Infinite M200, Tecan, Switzerland). To allow comparison of the results, the value of each group was divided by the value of the day 1-vehicle group and then multiplied by 100% for normalization.

### Alkaline phosphatase (ALP) staining and ALP activity assay

After osteogenic induction, hPDLSCs seeded in 12-well plates were fixed with 10% formaldehyde for 10 min. Then, the cells were incubated with BCIP/NBT Alkaline Phosphatase Color Development Kit (C3206; Beyotime, China) solution for 10–30 min until the appropriate staining color was observed. For the ALP activity assay, after osteogenic induction, hPDLSCs were harvested with cold Tris–HCl (50 mM, pH 7.4) and then sonicated for 20 s to lyse the cells. After centrifugation at 10,000 rpm at 4 °C for 20 min, ALP activity was measured using an alkaline phosphatase assay kit (P0321M; Beyotime). The total protein concentration in each sample was determined using the Detergent Compatible Bradford Protein Assay Kit (P0006C; Beyotime).

### Alizarin staining and quantitative analysis

hPDLSCs undergoing osteogenic differentiation were successively fixed with 4% formaldehyde and 95% ethanol for 10 min. Subsequently, the cells were incubated with alizarin red staining (A5533; Sigma, USA) solution (10 mg/mL) for 10 min and gently washed with distilled water 2–3 times to terminate the reaction. To quantify the results, stained cells were incubated with 1 mL of 10% hexadecylpyridinium chloride monohydrate (H108697; Aladdin) for 30 min to dissolve the mineralized nodules. Then, the absorbance of the dissolved solution was measured at 562 nm.

### Oil Red O staining

hPDLSCs undergoing adipogenic differentiation were fixed with 4% paraformaldehyde for 30 min and then stained with Modified Oil Red O Staining Kit (C0158S; Beyotime) solution for 10 min.

### Western blotting

Protein samples were separated by SDS‒PAGE and transferred onto polyvinylidene fluoride (PVDF) membranes (IPVH00010; Merck, Germany). The membranes were blocked with QuickBlock™ Blocking Buffer and incubated with QuickBlock™ Primary Antibody Dilution Buffer (P0239, Beyotime) and diluted Osterix (ab209484; Abcam, USA), Runx2 (A11753; ABclonal, China), OPN (A21084; ABclonal), OCN (A20800; ABclonal), Col-1 (14695-1-AP; Proteintech, USA), MFN1 (A21293; ABclonal), MFN2 (A19678; ABclonal), OPA1 (66583-1-Ig; Proteintech), DRP1 (A21968; ABclonal), MFF (A8700; ABclonal), FIS1 (A19666; ABclonal) and β-actin (66009-1-Ig; Proteintech) antibodies at 4 °C overnight. Then, the membranes were washed with PBS-Tween and incubated with DyLight 680 or 800 secondary antibodies (A23710, A23920; Abbkine, China) for 1 h. The stained protein bands were visualized using an Odyssey® DLx imaging system (LI-COR, USA). Fiji software was used to quantify band intensities.

### Cell sheet preparation and hematoxylin and eosin (H&E) staining

hPDLSCs were cultured in osteogenic differentiation medium with or without GOQDs in 6- or 12-well plates for 7 days. The cells at the bottom of the well exhibited a membranous structure and grew by curling toward the edges of the well. Then, the hPDLSC sheets were carefully separated and gently scraped by using microforceps and cell scrapers. The collected cell sheets were fixed with 4% paraformaldehyde for 24 h, subjected to gradient dehydration, immersed in paraffin, embedded, and sliced. H&E staining was then performed, and the sections were observed under a microscope. Five equally spaced straight lines at 45-degree angles were drawn on the captured photograph, and the width of the intersection between the lines and the cell sheets was measured using Fiji.

### Cell sheets, GOQDs, and GelMA mixture preparation

First, 0.05 g of the photoinitiator lithium phenyl-2,4,6-trimethylbenzoylphosphinate (LAP) (0.25 w/v) was dissolved in PBS at 40–50 °C for 15 min. The GelMA precursor solution (6% or 12% w/v) was prepared by dissolving 0.6 g or 1.2 g of sponge-form GelMA (EFL-GM-PR-002; Engineering for Life, China) in a 10-mL photoinitiator. To prepare the GOQD/GelMA mixed solution, 10 μL of GOQDs (1 mg/mL) was mixed well with 1.99 mL of GelMA precursor solution. After osteogenic induction, the hPDLSC sheets were cut into small pieces and mixed with GelMA precursor solution or GOQD/GelMA mixture. The mixture was placed in a rectangular mold (5 mm × 4 mm × 1.5 mm) and cured for 25 s or 50 s with 405 nm light. Afterward, the mixture was cultured in growth medium or osteogenic differentiation medium for the corresponding time. Then, the mixture was placed under a microscope for imaging; subjected to CCK-8, ALP, alizarin, and fluorescence staining; or transplanted into the mandibular bone defect area in rats.

### Scanning electron microscopy (SEM) observation of the hydrogel mixtures

GelMA or GelMA encapsulated with cell sheets was washed with PBS 3 times and fixed with 4% paraformaldehyde for 2 h. Afterward, the hydrogel or hydrogel mixture was incubated at − 20 °C for 24 h and frozen at − 80 °C for 1–2 h. The frozen hydrogel or hydrogel mixture was placed in a freeze-dryer for 24 h and then sprayed with gold. The pore structure of hydrogels with different concentrations of GelMA and the morphology of the cells were observed through SEM (SU3500, HITACHI, Japan).

### Fluorescence staining

Cell sheets, GOQDs, and GelMA mixtures were cultured in osteogenic differentiation medium for 14 days in 12-well plates. The mixture was fixed with 0.4% paraformaldehyde for 10 min and incubated with 0.1% Triton X-100 for 5 min. Then, the mixture was incubated with an anti-Col-1 primary antibody (A22090; ABclonal) for 2 h and an Alexa Fluor 488 goat IgG secondary antibody (K1206; APExBIO) for 30 min. Afterward, the mixture was sequentially treated with 1% v/v Actin-Tracker Red-Rhodamine (C2207S; Beyotime) for 30 min and DAPI for 4 min. The stained mixture was examined using an inverted fluorescence microscope (Nikon, Japan).

### Construction of periodontal bone defect models and implantation of hydrogel mixtures in rats

Animal studies were performed in accordance with the guidelines approved by the Institutional Animal Care Committee of China Medical University (CMU2021354). Male Sprague–Dawley (SD) rats (12 weeks old; weight, 250–300 g) were randomly divided into five groups (n = 6): (1) empty, no materials were implanted; (2) GelMA, GelMA hydrogels were implanted; (3) Vehicle/Cell/GelMA, GelMA was implanted with osteogenic differentiated hPDLSC-cell sheet fragments; (4) Y-GOQDs/Cell/GelMA, Y-GOQDs and GelMA were implanted with Y-GOQD-induced osteogenic differentiated-hPDLSC-cell sheet fragments; and (5) B-GOQDs/Cell/GelMA, implantation of B-GOQDs and GelMA were implanted with B-GOQD-induced osteogenic differentiated-hPDLSC-cell sheet fragments. Rats were subjected to adaptive feeding for one week, followed by intraperitoneal anesthesia using Zoletil® 50 (Virbac, France) and xylazine (Huamu Animal Health Products Co., Ltd., China). A skin incision was made along the oral fissure direction at the upper border of the mandible, followed by blunt separation of the subcutaneous tissue and the muscle layer to expose the bone surface. A bone defect with dimensions of approximately 5 × 4 × 1 mm was prepared at the root surface of the first and second molars using a dental turbine. The defect was located 0.5 mm beneath the crest of the alveolar bone and 1 mm away from the starting point of the masseter muscle fibers. Intraoperatively, the wound was washed with sterile saline and wiped with gauze. Then, the hydrogel mixtures were implanted according to their respective groupings. The muscle layer and skin were sutured in a layered and aligned manner.

### Micro-CT and histological detection of bone defects

Rats were euthanized at 2 and 4 weeks postsurgery, and the mandibles were collected and fixed with 4% paraformaldehyde. Micro-CT scans were performed at an isotropic voxel size of 20 μm. Three-dimensional reconstruction of the mandible was performed with CTvox software, the bone volume (BV), BV/tissue volume (TV), and bone surface density and was analyzed with CTAn software. All the samples were subsequently demineralized for at least 4 weeks, embedded, dehydrated, sectioned, and stained with H&E or Masson’s trichrome.

### Transmission electron microscopy (TEM) observation of mitochondrial morphology

hPDLSCs were cultured in osteogenic differentiation medium with or without GOQDs for 7 days. The cells collected by enzymatic digestion were stored in a TEM fixative solution (G1102; Servicebio®, China) at 4 °C. Next, the cells were fixed with 1% osmium tetroxide, dehydrated with an ascending ethanol series, infiltrated with propylene oxide, embedded in epoxy resin, sliced (90-nm thick), stained with lead citrate, examined via TEM (H-7650, HITACHI) and photographed.

### Active mitochondria staining and mitochondrial length analysis

hPDLSCs were cultured in osteogenic differentiation medium with or without GOQDs in glass bottom dishes for 7 days. The cells were subsequently treated with 200 nM MitoTracker Green (C1048; Beyotime) and 1% v/v Hoechst 33342 (C1028; Beyotime) for 30 min each at 37 °C. After washing with HBSS, α-MEM was added to the dishes. The stained cells were examined under a confocal microscope to obtain images of the mitochondria. Statistical analysis of mitochondrial networks was performed on three or more intact cells in each group. Mitochondrial branch lengths and footprints were analyzed using MiNA 3.0 (https://github.com/StuartLab/MiNA), a Fiji plugin for mitochondrial morphology analysis.

### Transfection of siRNA

MFN2 siRNAs (siMFN2) and negative control siRNA (siNC) were designed and synthesized by GenePharma (China). The targeting sequence for siMFN2 was 5’-CGGTTCGACTCATCATGGA-3’. Transfection was performed using jetPRIME® (101000046; PolyPlus, France). Briefly, 2 µg of siMFN2 was diluted in 100 µL of jetPRIME® buffer. After vortexing for 10 s, 1.5 µL of jetPRIME® was mixed and incubated for 10 min at room temperature. When the cells in the plate proliferated to 70–80% confluence, the medium was changed to 900 µL of osteogenic differentiation medium containing the above transfection mixture. Transfection was performed every three days.

### Statistical analysis

The data are presented as the mean ± standard deviation (SD). Unless otherwise specified, Tukey’s test was applied for multiple comparisons following one-way or two-way analysis of variance (ANOVA) for the data to compare the means of three or more groups. *P < 0.05; **P < 0.01; ***P < 0.001; ****P < 0.0001; NS, not significant (*P* > 0.05). Three biological replicates were used for each experiment, and representative images are shown in the figures.

## Results

### Characteristics of hPDLSCs

**Fig. 1:**
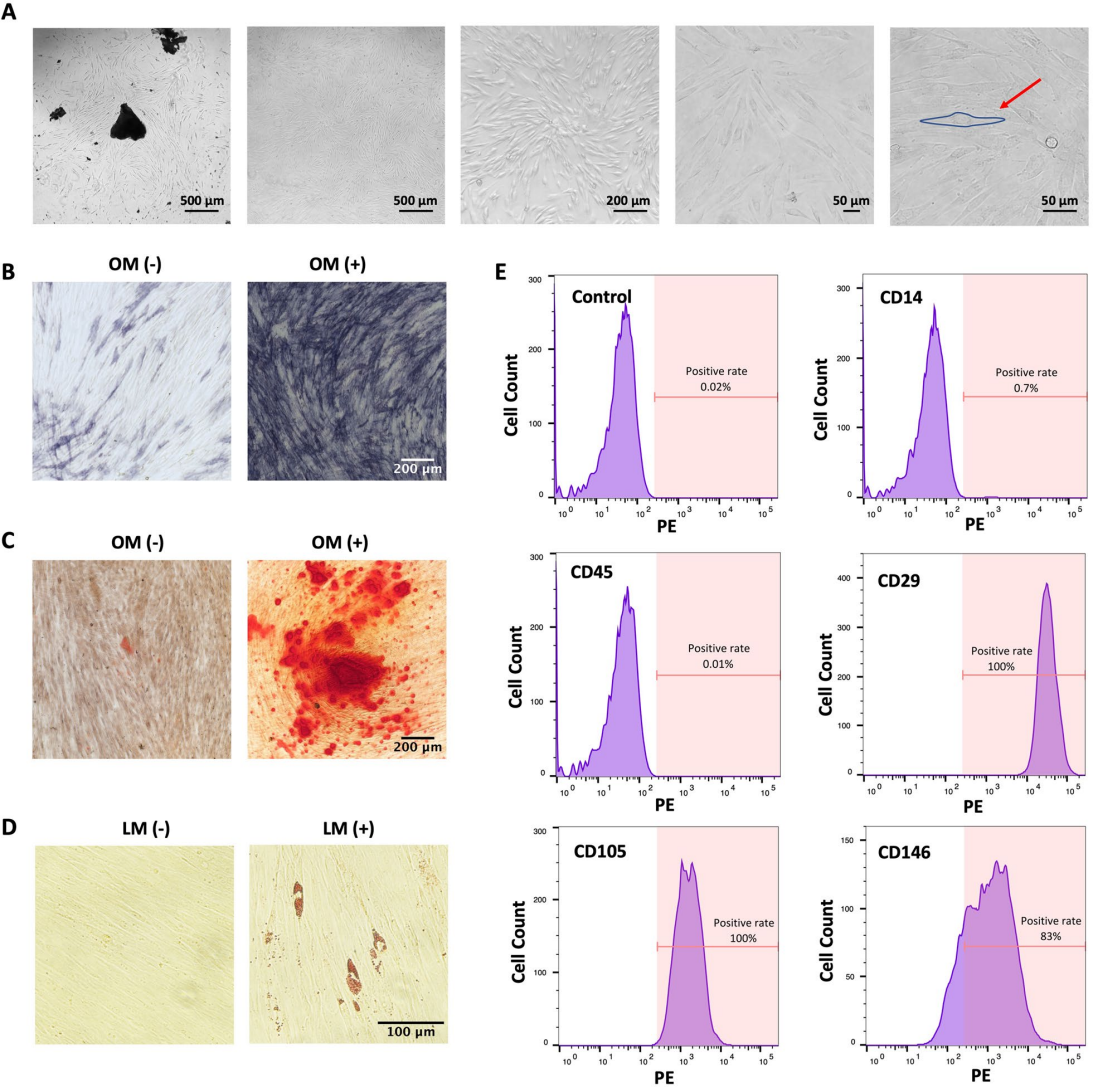
Identification and characteristics of hPDLSCs. **A** The spindle-shaped morphology of extracted hPDLSCs. **B**, **C**, **D** Representative images of the multilineage differentiation potential of hPDLSCs. ALP staining after 14 days (**B**) and Alizarin Red staining after 21 days (**C**) were used to assess osteogenic differentiation. Oil Red O staining after 21 days to assess adipogenic differentiation (**D**). **E** Flow cytometry analysis was used to identify the surface markers of hPDLSCs to confirm their mesenchymal stem cell characteristics

hPDLSCs migrated away from the periodontal ligament tissue block and formed vortex-like colonies. Under high magnification, the hPDLSCs exhibited a long fusiform spindle-shaped morphology (red arrow) (Fig. 1A). When cultured in osteogenic medium (OM), hPDLSCs exhibited a deep color from ALP staining (Fig. 1B) and Alizarin Red S-stained mineralized nodules were observed (Fig. 1C), indicating that hPDLSCs can differentiate into osteogenic cells. However, the hPDLSCs differentiated into adipogenic cells and formed lipid droplets in adipogenic medium (AM) (Fig. 1D). Flow cytometry analysis revealed that hPDLSCs were positive for the MSC markers CD29, CD105 and CD146 but negative for the hematopoietic markers CD14 and CD45 (Fig. 1E). These data demonstrated that the hPDLSCs were mesenchymal-derived cells and exhibited multidirectional differentiation potential.

### Characteristics of the GOQDs

**Fig. 2:**
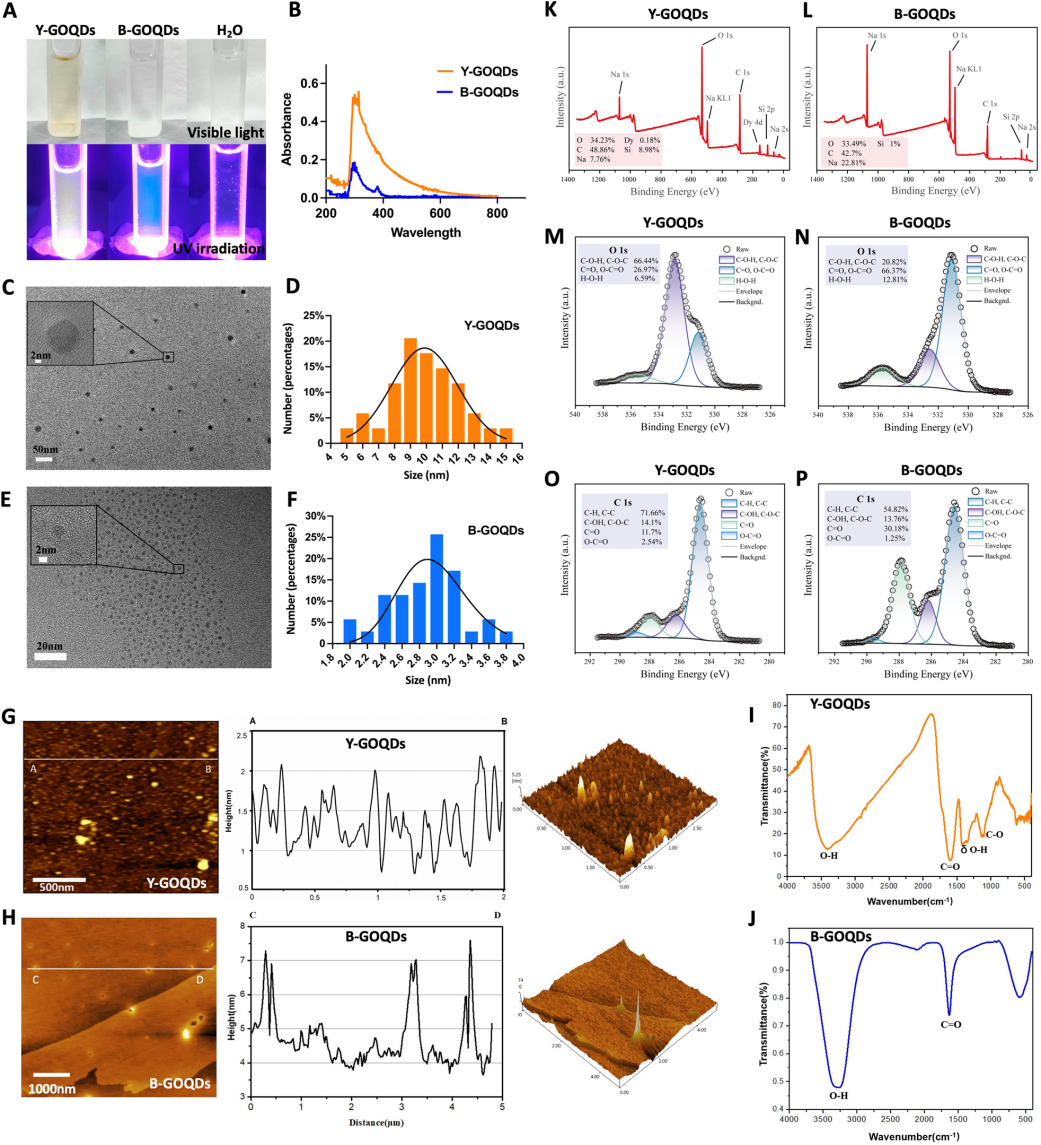
Characteristics of the GOQDs. **A** Photographs of Y-GOQDs, B-GOQDs, and pure water in sunlight and UV light. **B** Y-GOQD and B-GOQD wavelengths were measured by a UV spectrophotometer. **C**, **D** HRTEM images and diameter distribution analysis of Y-GOQDs. **E**, **F** HRTEM images and diameter distribution analysis of B-GOQDs. **G**, **H** AFM images and thickness distribution analysis of Y-GOQDs and B-GOQDs, respectively. **I**, **J** Fourier transform infrared spectra of Y-GOQDs and B-GOQDs. **K**, **L** The peak shapes of the main elements of Y-GOQDs and B-GOQDs, respectively. **M**, **N** O 1 s spectra of Y-GOQDs and B-GOQDs, respectively. **O**, **P** C 1 s spectra of Y-GOQDs and B-GOQDs, respectively

Y-GOQDs and B-GOQDs, fabricated using two synthesis methods, were characterized and assessed to determine differences in their physical structure and chemical composition. Y-GOQD and B-GOQD aqueous solutions had excellent dispersibility without any precipitation at the bottom; pure water was used as the control (upper plot, Fig. 2A). The photoluminescence properties of these materials varied: light fluorescent yellow for Y-GOQDs and fluorescent blue for B-GOQDs under 405 nm photoirradiation (bottom plot, Fig. 2A). UV‒visible absorption analysis revealed absorption peaks at 300 nm for Y-GOQDs and B-GOQDs, and the absorption data indicated that Y-GOQDs had a greater ability to absorb visible light (Fig. 2B). Microphotographs and an analysis of the diameter distribution are shown in Fig. 2C–F. The average diameter was approximately 9.95 nm for Y-GOQDs and 2.89 nm for B-GOQDs. AFM measurements revealed average thicknesses of ~ 1.36 nm for Y-GOQDs and ~ 3.59 nm for B-GOQDs (Fig. 2G–H). Considering the average height of the graphene layer is ~ 1.2 nm, Y-GOQDs usually consist of a single graphene oxide sheet, and B-GOQDs have 3 ~ 5 layers.

The chemical structures of Y-GOQDs and B-GOQDs were characterized by FTIR and XPS. The FTIR spectrum showed that the Y-GOQDs contained hydroxyl groups (–OH, γO–H≈3407.87 cm^-1^, δO–H≈1423.16 cm^−1^), carbonyl groups (C=O, γC=O≈1595.29 cm^−1^) and epoxy groups (C–O, γC–O≈1109.02 cm^−1^) (Fig. 2I). The B-GOQDs only contained hydroxyl groups (O–H≈3282.65 cm^−1^) and carbonyl groups (C=O≈1636.33 cm^−1^) (Fig. 2J). To determine the content of oxygen-functional groups in the Y-GOQDs and B-GOQDs, XPS was performed. The O 1 s spectra of Y-GOQDs and B-GOQDs were visible, with three peaks at 531.1–531.8, 532.3–533.3 and 535.5–536.1 eV, representing the presence of C=O, O–C=O, C–O–H, C–O–C and H–O–H bonds, respectively (Fig. 2K–L). The high-resolution XPS spectrum of O 1 s exhibited sharp peaks at 532.9 eV corresponding to C–O–H and C–O–C bonds (66.44%) for Y-GOQDs (Fig. 2M) and a signal corresponding to C=O and O–C=O bonds (66.37%) at 531.1 eV in the B-GOQD sample (Fig. 2N). The C 1 s spectra of the two GOQDs were deconvoluted into four peaks: C–C bonds, C=O bonds, C–OH and C–O–C bonds, and O–C=O bonds. The high peaks for C–C bonds in Y-GOQDs and B-GOQDs represent typical *sp*^*2*^ structures. The peak at 286.26 eV was assigned to C–OH and C–O–C bonds (14.1%) for Y-GOQDs, and the peak at 287.9 eV (30.18%) was assigned to C=O bonds in B-GOQDs (Fig. 2O–P). The above results indicated that the atomic ratios of C–O–H and C–O–C bonds to C=O bonds were significantly different in the obtained samples, with a high concentration of hydroxyl groups and epoxy groups on the Y-GOQD surface; for B-GOQDs, there was a predominance of carbonyl groups.

### GOQDs promote the osteogenic differentiation of hPDLSCs in vitro

**Fig. 3:**
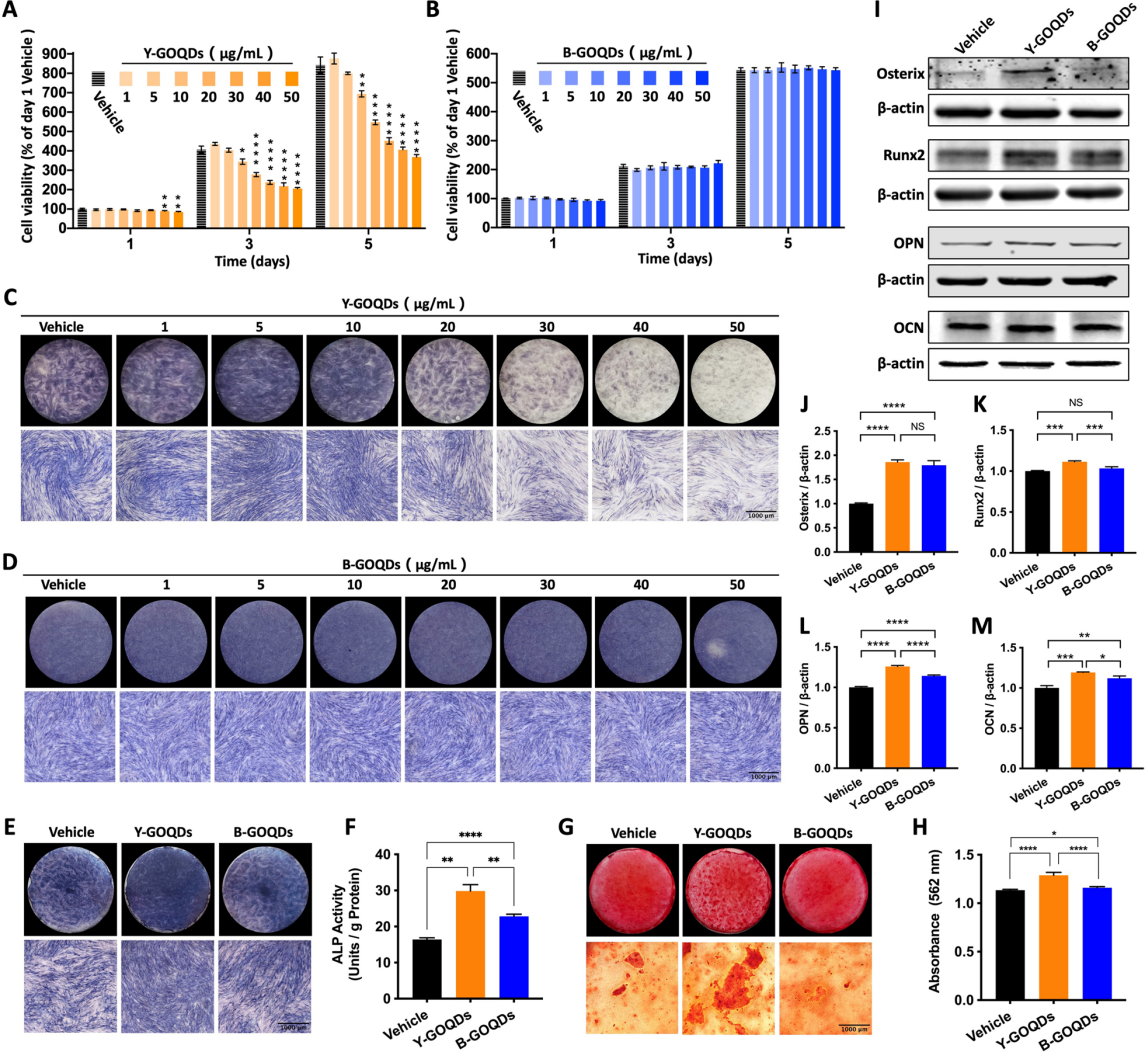
Effect of GOQDs on the viability and osteogenic differentiation of hPDLSCs in vitro. **A**, **B**) Cell viability assessed by the CCK-8 assay of hPDLSCs treated with different concentrations of Y-GOQDs (*n* = 6) or B-GOQDs (*n* = 5) for 1, 3 and 5 days. “*” represents a comparison of differences with the vehicle group at each time point. **C**, **D** ALP staining of hPDLSCs after osteogenic induction for 10 days with different concentrations of Y-GOQDs and B-GOQDs. **E**, **F** ALP staining and ALP activity (*n* = 4) of hPDLSCs after incubation with 5 µg/mL Y-GOQDs or B-GOQDs for 10 days. **G**, **H** Alizarin red staining and quantitative analysis of hPDLSCs after incubation with Y-GOQDs and B-GOQDs for 21 days. **I** Western blotting was used to assess the expression of osteogenic differentiation marker proteins, including Osterix, Runx2, OPN, and OCN, in hPDLSCs treated with Y-GOQDs and B-GOQDs. **J**, **K**, **L**, **M** Relative quantitative analysis of the protein expression of Osterix, Runx2, OPN, and OCN determined by Fiji (*n* = 3). The vehicle group was the solvent control group. NS* P* > 0.05, *P < 0.05, **P < 0.01, ***P < 0.001, ****P < 0.0001

To ensure that the GOQDs are safe for cell culture, we first evaluated the effect of the GOQDs on hPDLSC viability. After 1, 3 and 5 days of incubation, the CCK-8 assay results showed that Y-GOQDs at concentrations less than 5 μg/mL (Fig. 3A) and B-GOQDs at concentrations less than 50 μg/mL (Fig. 3B) had no significant effect on hPDLSC viability. Then, ALP staining was used to verify whether the GOQDs regulate the osteogenic differentiation of hPDLSCs. The results showed that 5–10 μg/mL Y-GOQDs significantly enhanced ALP staining (Fig. 3C), but none of the concentrations of B-GOQDs seemed to enhance ALP staining (Fig. 3D). Compared with the vehicle group, the phase-contrast images showed that Y-GOQDs at concentrations of 1–10 μg/mL and all concentrations of B-GOQDs had no significant effect on cell density or cell morphology. Moreover, the number of contracted dead cells (yellow arrows) increased and the cell density decreased with increasing Y-GOQDs concentration above 20 μg/mL (Additional file 1: Fig. S1). Considering both their biosafety and osteoinductivity, Y-GOQDs and B-GOQDs at a concentration of 5 μg/mL were used in subsequent experiments. Next, the ability of the two GOQDs to promote osteogenic differentiation was compared. As shown in the results, for hPDLSCs cultured in osteogenic medium, Y-GOQDs significantly promoted ALP activity (Fig. 3E, F), the formation of Alizarin Red S-stained mineralized nodules (Fig. 3G, H), and the protein expression of osteogenic differentiation markers, including Osterix, Runx2, OPN, and OCN (Fig. 3I–M). In contrast, the capacity to promote osteogenic differentiation was significantly weaker for the B-GOQDs than for the Y-GOQDs (Fig. 3E–M). Taken together, these findings indicate that Y-GOQDs are more effective than B-GOQDs at promoting the osteogenic differentiation of hPDLSCs in vitro.

### Construction and characterization of GOQD-treated cell sheet-laden GelMA in vitro

**Fig. 4:**
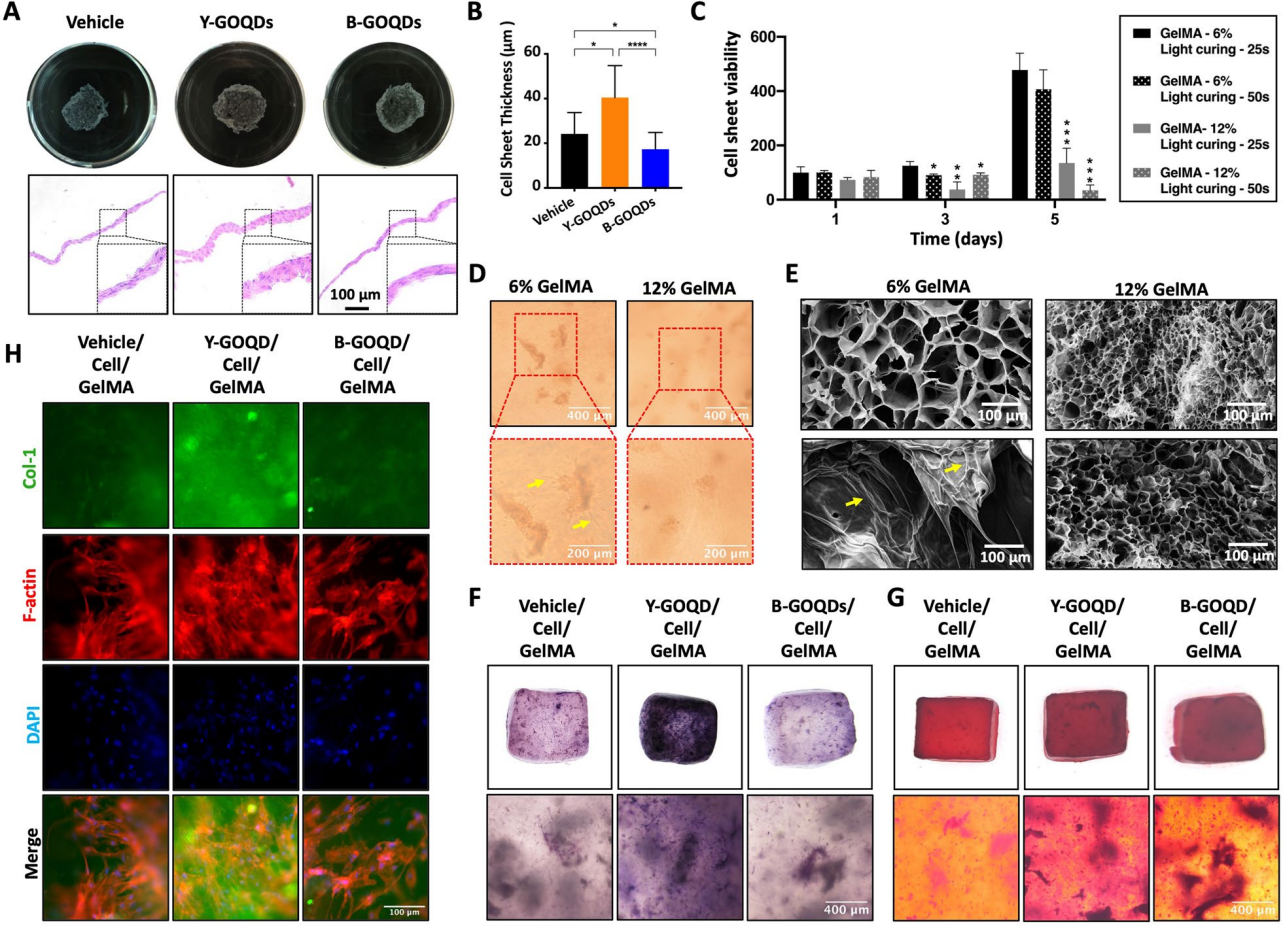
Construction and characterization of GOQD-treated cell sheet-laden GelMA in vitro. **A** Naked-eye views and H&E staining images of hPDLSC-cell sheets after osteogenic induction with/without GOQDs for 7 days. **B** Thickness measurements of cell sheets through Fiji were performed and analyzed by the Kruskal‒Wallis test (*n* = 3). **C** Cell activity of hPDLSC-cell sheet fragments encapsulated in different concentrations of hydrogels after different curing times (*n* = 4). **D** Microscopic observation of the cell growth state after packaging hPDLSC-cell sheet fragments with different concentrations of GelMA hydrogels for 24 h. **E** SEM images. The upper images show cross-sectional views of 6% and 12% w/v GelMA hydrogels without cell sheets after curing. The lower images display details of the encapsulated hPDLSC-cell sheets in 6% and 12% w/v GelMA hydrogels for 3 days. F, G, H) GOQD-treated cell sheet fragment-laden GelMA was induced in osteogenic medium with/without GOQDs in vitro: **F** ALP staining after 10 days; **G** Alizarin red staining after 21 days; **H** Immunofluorescence staining of Col-1 and F-actin after 14 days. The vehicle group was the solvent control group. * *P* < 0.05, ** *P* < 0.01, *** *P* < 0.001, **** *P* < 0.0001

To construct a more active and collagen-rich cell-scaffold complex, we used cell sheets instead of cells. After 7 days of osteogenic induction, cell sheets had formed from hPDLSCs cultured with/without GOQDs and successfully separated from the plate bottom. The cell sheets of hPDLSCs treated with Y-GOQDs or B-GOQDs were light yellow or white transparent, respectively (upper plot, Fig. 4A). H&E staining revealed that vehicle-cell sheets and B-GOQD-cell sheets consisted of 2–3 layers of cells but that Y-GOQD-cell sheets had 3–4 or more layers with a robust extracellular matrix (ECM) (bottom plot, Fig. 4A). The thickness of the cell sheets measured by Fiji revealed that the Y-GOQD-cell sheets were thicker than the other cell sheets, with no obvious differences between the vehicle and B-GOQD-cell sheets (Fig. 4B). Then, we sliced the GOQD-untreated cell sheets into fragments, loaded them into hydrogels with different GelMA concentrations and treated them for different durations. The cell sheet viability experiment revealed that 6% w/v GelMA with a 25 s UV curing time was the most suitable growth condition for cell sheet fragments (Fig. 4C). Many cells spread out from the cell sheet fragments encapsulated in 6% w/v GelMA after a 25 s curing time, but a similar phenomenon was not observed for 12% w/v GelMA (Fig. 4D). According to the cross-sectional SEM images, the 6% w/v GelMA hydrogels had a loose network porous structure with a large pore size; comparatively, the structure of the 12% w/v GelMA hydrogel was dense with small pores (upper plot, Fig. 4E). After culture in growth media for 3 days, the porous structure was squeezed by the growing cell sheets and began to degrade in 6% w/v GelMA with cell sheet fragments (yellow arrows); however, it is difficult to distinguish the cell sheets from the hydrogels in the SEM images. In contrast, in the 12% w/v GelMA with cell sheet fragments, due to the poor growth of the cell sheets, the porous structures of hydrogels remained relatively intact (bottom plot, Fig. 4E). In brief, 6% w/v GelMA cured for 25 s maintained good growth of cell sheet fragments in vitro.

We subsequently compared the osteogenic capacity of GOQD-treated and untreated cell sheet-laden GelMA in vitro. After osteogenic induction with Y-GOQDs or B-GOQDs for 7 days, cell sheets of hPDLSCs were obtained. The GOQD-pretreated cell sheet fragments were then loaded into 6% w/v GelMA together with Y-GOQDs or B-GOQDs to prepare GOQD-treated cell sheet-laden GelMA. Then, the cell sheet-laden GelMA was continuously induced with osteogenic medium for another 10 or 21 days. The most intense ALP (Fig. 4F) and Alizarin staining (Fig. 4G) were observed in the Y-GOQD-treated cell sheet-laden GelMA group compared with the vehicle and B-GOQD groups. The expression of Col-1, considered the major component protein in the ECM, was also increased in the Y-GOQD-treated cell sheet-laden GelMA group (Fig. 4H). These results demonstrated that, compared with B-GOQDs, Y-GOQDs more effectively promoted the osteogenic differentiation of cell sheet fragments in the scaffold of GelMA hydrogels.

### GOQD-treated cell sheet-loaded GelMA facilitates periodontal bone defect repair in vivo

**Fig. 5:**
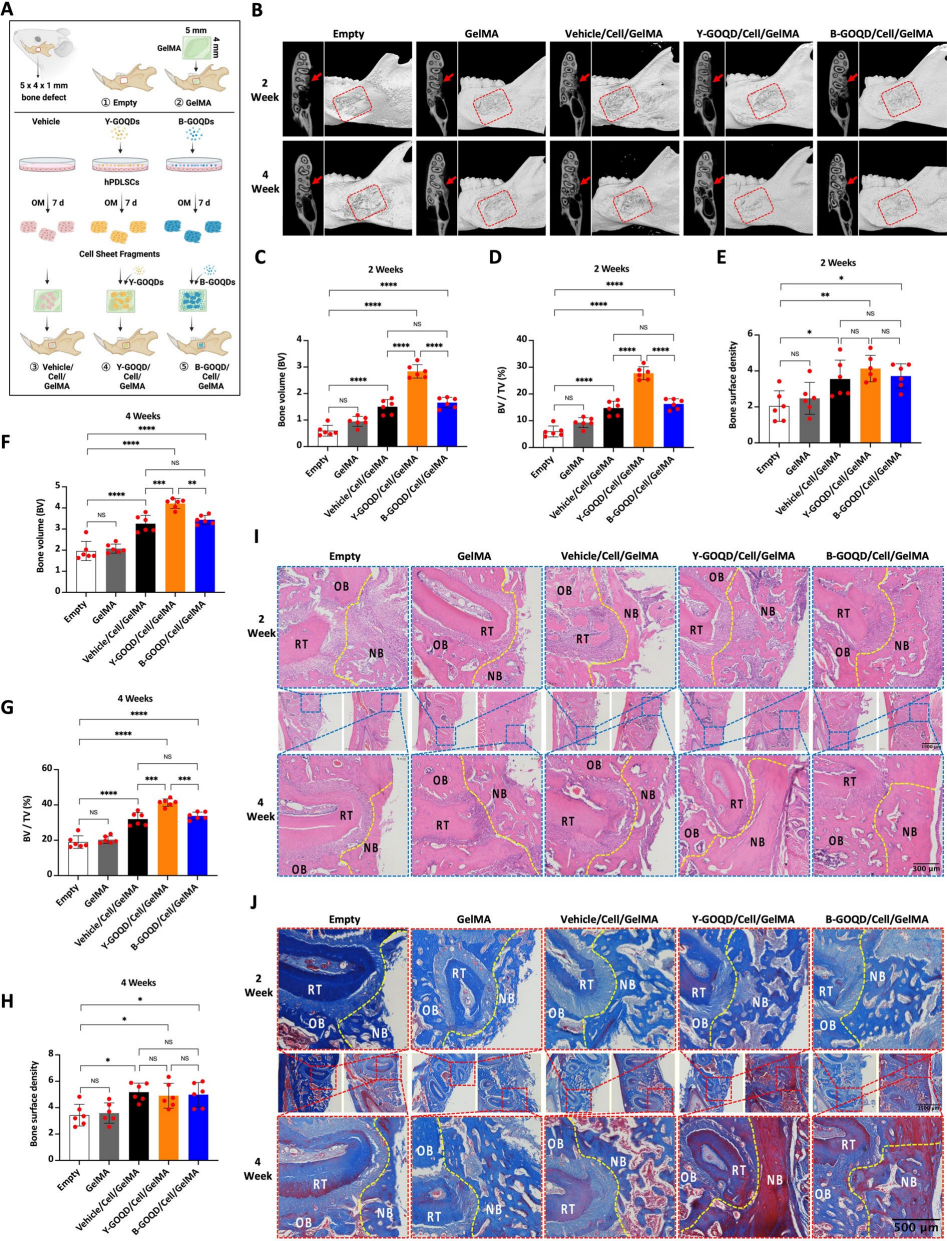
GOQD-treated cell sheet-loaded GelMA facilitates periodontal bone defect repair in vivo. **A** Schematic diagram of the rat periodontal fenestration defect experiment. **B** Micro-CT cross-sectional and three-dimensional reconstruction of mandibular periodontal bone defects at 2 weeks and 4 weeks after surgery. **C**–**H** Comparisons of bone volume (BV) (**C**, **F**), bone volume fraction (BV/TV) (**D**, **G**) and bone surface density (**E**, **H**) at 2 weeks and 4 weeks (*n* = 6). **I**, **J** Images of HE staining (**I**) and Masson’s trichrome staining (**J**) of mandibular bone defects at 2 weeks and 4 weeks. *OB* old bone, *NB* new bone, *RT* root. NS* P* > 0.05, * *P* < 0.05, ** *P* < 0.01, *** *P* < 0.001, **** *P* < 0.0001

To evaluate the effects of bone regeneration in vivo, nothing (empty), GelMA (GelMA), GOQD-untreated cell sheet-laden GelMA (Vehicle/Cell/GelMA), or GOQD-treated cell sheet-laden GelMA (Y-GOQDs/Cell/GelMA, B-GOQDs/Cell/GelMA) hydrogels were implanted into mandibular periodontal bone defects of rats for 2 or 4 weeks (Fig. 5A). The entire surgical procedure is shown in Additional file 2: Fig. S2. Micro-CT and 3D reconstructed images were used to visualize the regeneration of new bone in the defect area. In the empty and GelMA groups, more obvious bone defects could still be observed in the surgical area. In contrast, bone regeneration and bone repair in the defect area in the Y-GOQD/Cell/GelMA and B-GOQD/Cell/GelMA groups were observed at different time points (red rectangles, Fig. 5B). Analysis of the new bone volume (BV) and new bone volume ratio (BV/TV) at 2 and 4 weeks revealed that the BV and BV/TV in the Y-GOQD/Cell/GelMA group were significantly greater than those in the other groups (Fig. 5C, D, F, G). There was no significant difference in the BV or BV/TV between the vehicle/cell/GelMA group and the B-GOQD/cell/GelMA group at 2 and 4 weeks (Fig. 5C, D, F, G). However, there were no significant differences in bone surface density among the vehicle/cell/GelMA group, the Y-GOQD/Cell/GelMA group, or the B-GOQD/Cell/GelMA group at 2 and 4 weeks (Fig. 5E, H). HE and Masson’s trichrome staining were also conducted to evaluate the quality and mass of the new bone. The results showed that the margin of the defect in the Empty group at 2 weeks was mainly filled with fibrous connective tissue. However, the Y-GOQD/Cell/GelMA group displayed more bone regeneration, and newly formed trabeculae were observed (upper plot, Fig. 5I). At 4 weeks, little woven bone formation was observed on the surface of the defect area in the Empty and GelMA groups, and the amount of newly formed bone in the Vehicle/Cell/GelMA, B-GOQD/Cell/GelMA and Y-GOQD/Cell/GelMA groups seemed to increase in turn (bottom plot, Fig. 5I). In addition, more mature bone with dark red staining was found at the margin and surface in the defect region of the Y-GOQD/Cell/GelMA group than in the blue-stained neonatal bone in the empty, GelMA and vehicle/cell/GelMA groups (Fig. 5J). H&E staining of organs in rats, such as the heart, liver, spleen, lungs and kidneys, revealed no significant differences in cell morphology between the treatment groups and the empty group; additionally, no organic lesions were observed in the treatment groups, indicating that the GOQD-treated cell sheet-laden GelMA has good biological safety in vivo (Additional file 3: Fig. S3). In summary, for large periodontal bone defects in vivo, Y-GOQD-treated cell sheet-laden GelMA showed enhanced bone regeneration and bone repair ability. B-GOQD-treated cell sheet-loaded GelMA also promoted the maturation of new bone.

### Y-GOQDs promote hPDLSC mitochondrial fusion and inhibit mitochondrial fission

**Fig. 6:**
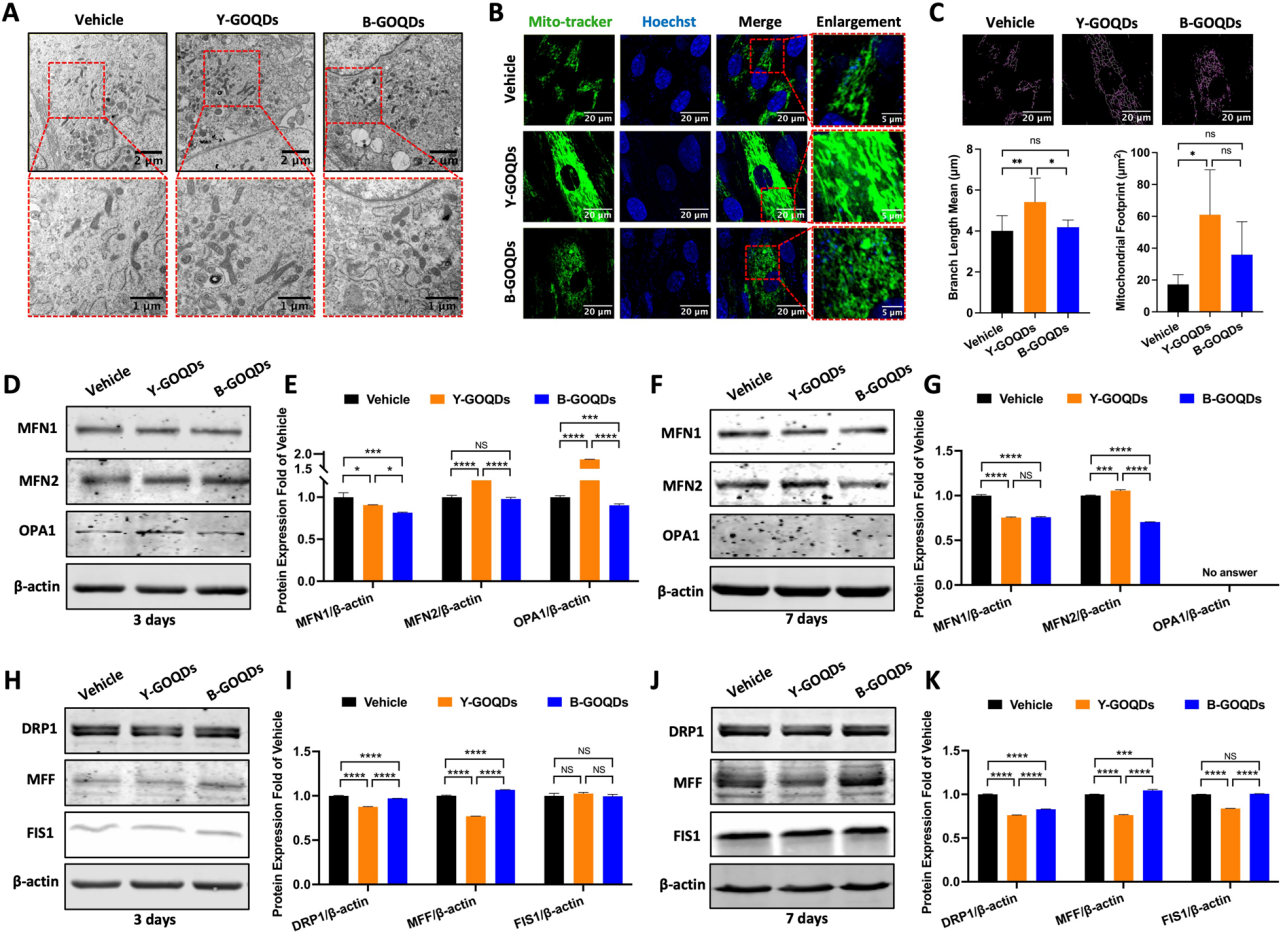
The effects of GOQDs on mitochondrial morphology and the expression of proteins regulating mitochondrial dynamics in hPDLSCs. **A** After 7 days of GOQD treatment, the mitochondrial morphology of hPDLSCs was observed via TEM. **B** After 7 days of GOQD treatment, morphological changes in the mitochondrial network were observed in hPDLSCs using laser confocal microscopy (MitoTracker Green). **C** Mitochondrial skeleton images obtained through Fiji software (upper plot) and statistical analysis of mitochondrial length and the mitochondrial footprint (bottom plot) by the Kruskal‒Wallis test (*n* ≥ 3). **D**, **E**, **F**, **G** Western blot analysis of the expression of mitochondrial fusion marker proteins (MFN1, MFN2, and OPA1) after GOQD treatment for 3 days (**A**) or 7 days (**C**). **E** and **G** Relative quantitative analyses of (**D**) and (**F**) (*n* = 3), respectively. **H**, **I**, **J**, **K**) Western blot analysis of the expression of mitochondrial fission marker proteins (DRP1, MFF, and FIS1) after GOQD treatment for 3 days (**H**) or 7 days (**J**). **I** and **K** Relative quantitative analysis of (**H**) and (**J**), respectively (*n* = 3). The vehicle group was the solvent control group. NS* P* > 0.05, *P < 0.05, **P < 0.01, ***P < 0.001, ****P < 0.0001

The above results showed that Y-GOQDs were more effective than B-GOQDs at promoting the osteogenic differentiation of hPDLSCs to repair bone defects. Subsequently, we further explored the biological mechanism behind this phenomenon. Mitochondrial morphology, as determined by TEM, revealed that the mitochondria in Y-GOQD-treated hPDLSCs were larger and longer than those in vehicle- and B-GOQD-treated hPDLSCs (Fig. 6A). The mitochondrial network was further examined after MitoTracker staining. After treatment with Y-GOQDs, the mitochondria of the hPDLSCs exhibited a dense network structure and strong high-density fluorescence (Fig. 6B). The mean branch length of mitochondria and the area occupied by mitochondria in those hPDLSCs also increased (Fig. 6C). In contrast, the mitochondria in the vehicle and B-GOQD groups exhibited a punctate morphology, and the length and area of the mitochondria were also not significantly different (Fig. 6B, C). The above results suggested that Y-GOQDs affect the mitochondrial dynamics, namely, mitochondrial fusion and fission, of hPDLSCs. To further confirm the activation of mitochondrial dynamics, the expression of mitochondrial fusion- and fission-related proteins was evaluated. After 3 days, the expression of the fusion-related proteins MFN2 and OPA1 significantly increased in Y-GOQD-treated hPDLSCs (Fig. 6D, E). On day 7, in Y-GOQD-treated hPDLSCs, the expression of MFN2 was also greater than that in the other groups (Fig. 6F, G). Similarly, compared with those in the vehicle group, both Y-GOQD- and B-GOQD-treated hPDLSCs exhibited a decrease in the expression of the fission-related proteins DRP1, MFF, and FIS1 on either day 3 or day 7 (Fig. 6H–K). Generally, Y-GOQDs promoted early mitochondrial fusion in hPDLSCs and inhibited mitochondrial fission, and B-GOQDs slightly inhibited mitochondrial fission.

### Knockdown of MFN2 attenuated the promoting effect of GOQDs on the osteogenic differentiation of hPDLSCs

**Fig. 7:**
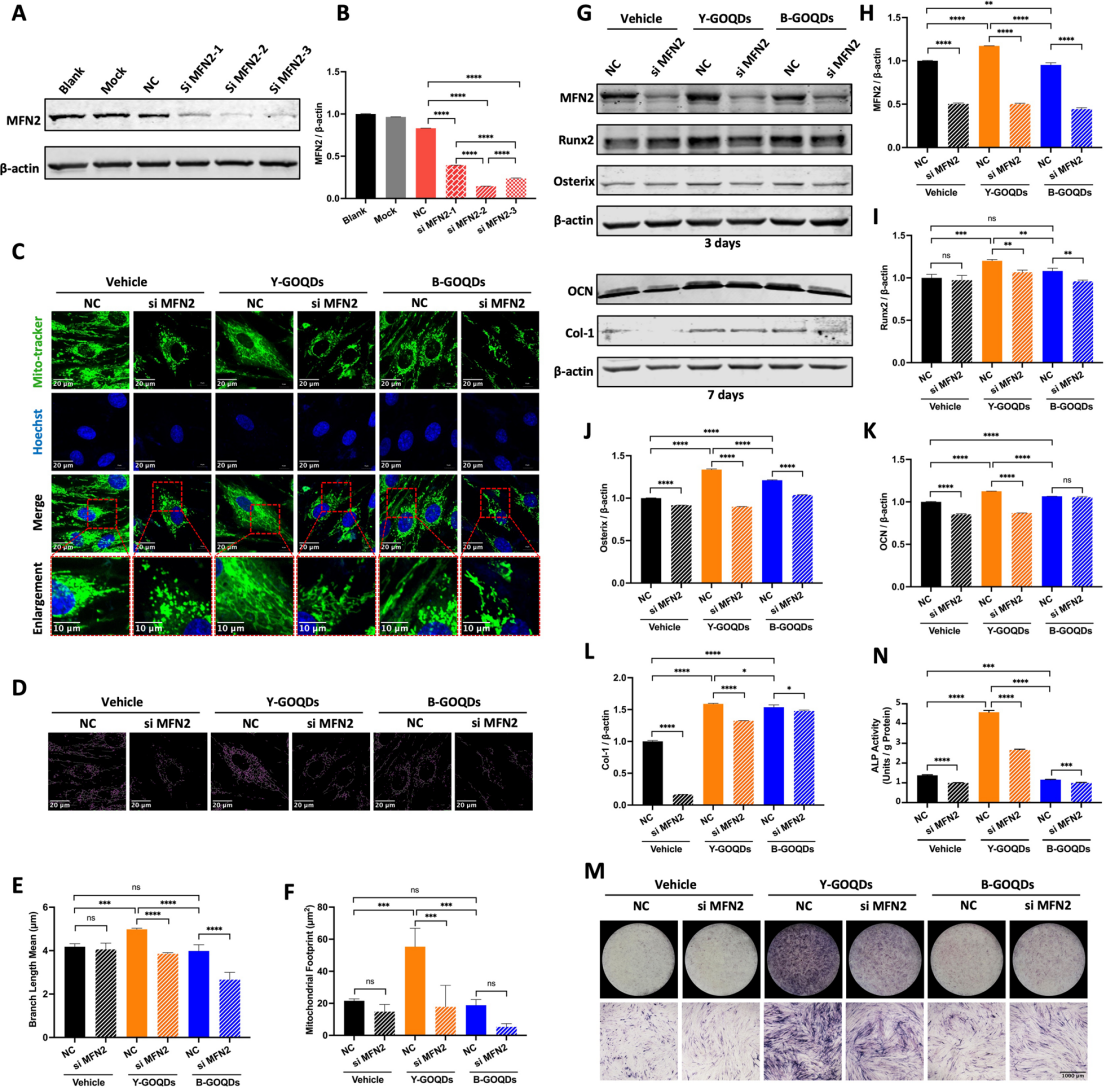
Knocking down of MFN2 attenuated the osteogenic differentiation of hPDLSCs promoted by the GOQDs. **A** Western blotting was performed to verify the most efficient MFN2 knockdown sequence 7 days after transfection. The blank group was not treated, the mock group was treated with transfection reagent, and the NC group was treated with NC-siRNA. **B** Relative quantification analysis of the data in (**A**) (*n* = 3). **C** hPDLSCs were treated with GOQDs for 7 days with or without MFN2 siRNA in osteogenic medium, and morphological changes in the mitochondrial network were observed via laser confocal microscopy (MitoTracker Green). **D** Mitochondrial skeleton images obtained through Fiji software. **E** Statistical analysis of mitochondrial length (*n* ≥ 3). **F** Statistical analysis of the mitochondrial footprint (*n* ≥ 3). **G** hPDLSCs were treated with GOQDs for 3 or 7 days with or without MFN2 siRNA in osteogenic medium, and the protein expression of MFN2, Runx2, Osterix, OCN, and Col-1 was assessed via Western blotting. **H**, **I**, **J**, **K**, **L**) Relative quantitative analysis of (**G**) (*n* = 3). **M**, **N** ALP staining and ALP activity (*n* = 4) assay of GOQD-treated hPDLSCs treated with or without MFN2 siRNA for 10 days in osteogenic medium. ns* P* > 0.05, * *P* < 0.05, ** *P* < 0.01, *** *P* < 0.001, **** *P* < 0.0001

Based on previous results, among the mitochondrial dynamics regulators, under Y-GOQD treatment, the expression of MFN2 was most significantly changed. Hence, we investigated whether knocking down MFN2 affects the mitochondrial dynamics and osteogenic differentiation of hPDLSCs treated with Y-GOQDs. Three sequences of MFN2 small interfering RNAs were designed, and we selected siMFN2-2, which had the highest knockdown efficiency (Fig. 7A, B). The knockdown of MFN2 caused the mitochondria of hPDLSCs treated with Y-GOQDs to change from a dense network to a loose short rod, and there was a similar trend in the Vehicle and B-GOQD groups (Fig. 7C). The length and area of the mitochondria in the Y-GOQD and B-GOQD groups also decreased significantly with the knockdown of MFN2 (Fig. 7D–F). Similarly, compared with that in the control (NC), the expression of the osteogenic differentiation markers Runx2, Osterix, OCN and Col-1 in the Y-GOQD-treated group was significantly lower (Fig. 7G–L). Similar trends were observed for ALP staining (Fig. 7M) and for ALP activity analysis (Fig. 7N). The above results revealed that knockdown of MFN2 attenuated the promoting effect of Y-GOQDs on the osteogenic differentiation of hPDLSCs. Therefore, Y-GOQDs promote the osteogenic differentiation of hPDLSCs by regulating mitochondrial dynamics.

## Discussion

With the progression of in-depth research on bone tissue regeneration, promoting the repair of bone tissue more effectively in the lesion area and restoring the original physiological structure and function have become popular topics in the field of bone regeneration medicine. In this study, we used hPDLSCs as seed cells and investigated the effects of two types of GOQDs prepared by two methods on the osteogenic differentiation of hPDLSCs in vitro. Subsequently, we constructed GOQD-treated hPDLSC cell sheet-loaded GelMA and verified its ability to repair rat periodontal bone defects in vivo. Finally, we explored the biological mechanism by which GOQDs promote the osteogenic differentiation of hPDLSCs through regulating mitochondrial dynamics.

The clinical application of stem cells is subject to various constraints, such as ethical and moral restrictions on umbilical cord MSCs and embryonic stem cells, donor trauma and limited source restrictions on bone marrow MSCs [35, 36]. hPDLSCs can be isolated from the periodontal ligament, possess the characteristics of MSCs, and can differentiate into osteoblasts or adipocytes. The results of our hPDLSC characterization experiments support the above findings. hPDLSCs are easy to obtain and simple to isolate. By analyzing whole-genome maps of hPDLSCs, dental pulp stem cells, and dental follicle progenitor cells, researchers have also found that hPDLSCs have increased transcription levels of osteogenic factors, increased in vitro osteogenic potential, and increased in vivo new bone formation ability [37]. Moreover, hPDLSCs have unique differentiation abilities, as they can differentiate into odontoblasts and bone-forming cells and are more likely to form “tooth-periodontal ligament-alveolar bone”-like structures in vivo, reconstructing attachments between periodontal tissues [5, 38]. For the above reasons, in this study, hPDLSCs were selected as the seed cells for repairing periodontal bone defects.

GOQDs are nanoscale derivatives of GO. They have been widely used in the field of biomedical science, such as biosensing, bioimaging, drug delivery, and photodynamic therapy [15, 18, 24, 39–42]. Recently, the application potential of GOQDs in tissue engineering has attracted our attention. In this study, Y-GOQDs and B-GOQDs were prepared by “top-down” and “bottom-up” methods, respectively. Y-GOQDs and B-GOQDs exhibit yellow and blue fluorescence, respectively, due to their different quantum sizes, which is consistent with the previously reported fluorescence characteristics of graphene quantum dots (GQDs) of different sizes [43]. Their fluorescence characteristics originate mainly from the peripheral carboxylic groups and conjugated carbon skeleton [43]. We compared the effects of Y-GOQDs and B-GOQDs on the viability and osteogenic differentiation of hPDLSCs. Y-GOQDs at concentrations greater than 10 µg/mL significantly inhibited the viability of hPDLSCs. Cell proliferation and differentiation have a remarkable inverse relationship [44]. The decrease in the hPDLSC proliferation rate after Y-GOQD treatment was not due to enhanced osteogenic differentiation, as we used growth medium without osteogenic inducer. Moreover, we observed that long-term exposure to high concentrations of Y-GOQDs led to cell damage characterized by vacuolization and shrinking. However, B-GOQDs at concentrations of 1–50 µg/mL had no significant effect on the viability of hPDLSCs throughout the observation period. Nanoparticle-mediated cytotoxicity appears to be governed by quantity of nanoparticle uptake, particle-cell interactions, particle physicochemical properties, or cell type [45]. Characterization of the GOQDs revealed that the Y-GOQDs had a flake-like structure with a diameter of approximately 10 nm and that the B-GOQDs had an approximately spherical structure with a diameter of approximately 3 nm. When flake-like Y-GOQDs pass through the cell membrane, they may contact the surface of the cell membrane in a face-to-face manner, and the friction force is high; moreover, spherical B-GOQDs may have point contacts with the cell surface, and these particles may be small in size; therefore, the friction force on the cell membrane is low. This observation supports the concept that the cytotoxicity of GO depends on its size [46]. In addition, compared to B-GOQDs, Y-GOQDs contain more oxygen-containing groups on the surface, readily causing oxidative stress damage to cells at high concentrations [47, 48]. Therefore, high concentration of Y-GOQDs have a relatively greater negative effect on the viability of hPDLSCs.

However, in the osteogenic differentiation experiment involving hPDLSCs treated with GOQDs, a completely different trend was observed. Y-GOQDs at concentrations ranging from 1 to 10 µg/mL significantly promoted the osteogenic differentiation of hPDLSCs, and B-GOQDs had only a weak osteogenic effect. Y-GOQDs contain more hydroxyl groups and C–O bonds, while B-GOQDs contain more C=O bonds. Compared with that for C=O, the bond length for C–O is longer, and the bond energy is lower; therefore, this covalent bond is relatively unstable [49]. Research has indicated that, after incubation in culture medium for 24 h, the GQD particle size increased from ~ 4.1 nm to 9.4–11.8 nm, indicating the adsorption of medium components [50]. Osteogenic differentiation media contains inducers such as dexamethasone, β-glycerophosphate, and ascorbic acid. The instability of the C–O bond endows it with a greater ability to bond, allowing it to readily bind to dexamethasone [51, 52]. Dexamethasone not only alters the expression levels of many osteogenic proteins and enzymes but also acts synergistically with β-glycerolphosphate and the intracellular enzyme alkaline phosphatase to synthesize new mineralized bone matrix [52]. Ascorbic acid is a cofactor for this enzyme, which triggers Col-1 secretion into the ECM [53]. The –OH moieties of ascorbic acid can form hydrogen bonds with the graphene substrate, and these hydrogen bonds can enhance the adhesion properties of ascorbic acid in the *sp*^*3*^ (C–O) carbon domains [52, 54]. The amount of ascorbic acid adsorbed onto GO increased 1.42-fold within 18 days [54]. These reasons may be why Y-GOQDs have a stronger osteogenic differentiation-promoting effect than B-GOQDs. Moreover, the size and morphology of the nanoparticles may all affect cell differentiation [55]. The flake-like Y-GOQDs are larger than the spherical B-GOQDs. On the one hand, Y-GOQDs have a larger specific surface area than B-GOQDs, so Y-GOQDs not only carry more C–O functional groups but also can adsorb more osteogenic inducers; on the other hand, the larger and flatter Y-GOQDs may possess more ligands on the surface and have a longer contact edge compared to B-GOQDs, endowing them with higher internalization efficiency. Cell receptor-mediated nanoparticle endocytosis efficiency depends on receptor diffusion kinetics, which includes the number of ligand-receptors and the contact edge length [45, 56]. However, in the future, if well-defined Y-GOQDs or B-GOQDs with different sizes are separated and purified by methods such as gel electrophoresis [43], the impact of the particle size of the GOQDs on osteogenic differentiation will be further elucidated. In brief, at relatively safe low concentrations, Y-GOQDs promoted the osteogenic differentiation of hPDLSCs more effectively than B-GOQDs did.

It is worth noting that Y-GOQDs at concentrations ranging from 20 to 50 µg/mL inhibited the osteogenic differentiation of hPDLSCs in a dose-dependent manner. This inhibition was due to cell damage caused by high concentration of Y-GOQDs. Previous studies have shown that Y-GOQDs at concentrations of 0.1–10 µg/mL significantly increased the intracellular ROS levels in *Microcystis aeruginosa* [57]. GOQDs (Same as Y-GOQDs) increased the physiological ROS level, which has a positive effect on osteogenic differentiation [20]. However, high concentrations of Y-GOQDs may likely cause an ROS burst in the cells due to the unstable C–O bond, ultimately hindering the osteogenic differentiation of hPDLSCs. B-GOQDs may have limited ROS-promoting effects due to the stable C=O bond; therefore, the B-GOQDs do not impact cellular activity or show an obvious osteogenic effect.

To increase the survival rate and osteogenic differentiation efficiency of hPDLSCs in vivo, we used cell sheet fragments instead of single cells. Cell sheet technology preserves autogenous ECM, intercellular junction proteins and functional proteins to the greatest extent by preventing cells from being digested with enzymes and can efficiently improve cell engraftment [58, 59]. Cell sheet technology can also prevent implantation-related inflammatory immune reactions, tissue collapse because of rapid degradation, and impaired tissue development in the context of slow scaffold degradation [60]. In this study, the porous structure of the GelMA hydrogel was selected as the basic scaffold material, which not only provides 3D growth space for cell sheet fragments but also maintains microenvironment stability and improves the activity of transplanted cells [61, 62]. Through the optimization of GelMA preparation conditions, we found that 6% GelMA formed large pores after UV curing for 25 s, which are the most suitable conditions for the growth of cell sheets. In addition, we incorporated the corresponding concentration of GOQDs into GelMA and used the slow release of the hydrogel to ensure that the encapsulated cell sheets continued to interact with the GOQDs even in vivo. Through SEM observation, we found that after 3 days of in vitro culture of 6% GelMA containing cell sheet fragments, the porous structure began to degrade, which could provide additional space for the growth of cell sheet fragments. However, the degradation rate of GelMA in vitro and in vivo needs to be further verified in future experiments. In vivo, we chose the mandibular first molar root as the study area for bone tissue regeneration. We prepared a rectangular critical bone defect that extended to the root. The lateral surface of the lower first molar root is relatively flat and has a large area, making it an ideal area for preparing critical bone defects. In addition, compared with those of the anterior teeth region, the histological characteristics of the mandibular molar region are more similar to those of human molars with peri-root bone defects. These results validate, for the first time, that Y-GOQD-treated cell sheet-laden GelMA can more efficiently repair periodontal bone defects and promote bone tissue regeneration in vivo. This also means that GOQDs, as low-cost and easily accessible nanosized quantum dots, have great potential for application in BTE.

A thorough understanding of the mechanism by which GOQDs promote osteogenic differentiation is crucial for the rational design of this material and for its application in BTE. Mitochondria not only are the main energy supply centers within the cell but also participate in the transmission of various cellular signals [63]. Mitochondrial metabolism is necessary for determining stem cell fate and plays an important regulatory role in the differentiation of MSCs [64, 65]. We observed subcellular organelles by TEM and found that Y-GOQD-treated hPDLSCs exhibited more elongated and well-defined functional mitochondria than did the vehicle and B-GOQD groups. This finding suggested that Y-GOQDs induce changes in mitochondrial dynamics in response to the energy metabolism demands of hPDLSC osteogenic differentiation. Fusion and fission are key events regulating mitochondrial dynamics. Mitochondrial fusion refers to the merging of two mitochondria into a single entity; this process is regulated by mitofusin-protein fusion of MFN1- and MFN2-triggered outer mitochondrial membrane fusion and OPA1-triggered inner mitochondrial membrane fusion. Mitochondrial fission refers to the division of a single mitochondrion into two steps, a process that is primarily carried out by cytoplasmic DRP1 binding to the outer mitochondrial membrane receptors MFF and FIS1 [66]. The results of our experiments demonstrated that Y-GOQDs mainly promoted mitochondrial fusion into a long network structure, accompanied by an increase in MFN2 and OPA1. The effect of B-GOQDs on mitochondrial fusion was not significant. Knocking down MFN2 significantly impaired the Y-GOQD-mediated osteogenic differentiation of hPDLSCs. These results demonstrated for the first time that the osteogenic effect of Y-GOQDs is achieved through the targeted regulation of mitochondrial dynamics. These results are supported by previous research indicating that during the differentiation of MSCs into osteogenic cells, energy acquisition shifts from glycolysis to oxidative phosphorylation, a process that requires the promotion of mitochondrial fusion to ensure the activity of the tricarboxylic acid cycle in mitochondria [65, 67, 68]. We speculate that the Y-GOQD-mediated regulation of mitochondrial dynamics may be due to the unique single-chain oxygen-containing groups of Y-GOQDs that promote upstream signals of mitochondrial fusion-regulating proteins. This topic requires further in-depth exploration in the future.

## Conclusion

In summary, GOQDs have abundant oxygen-containing functional groups, which give them excellent dispersibility and active biological properties. The nanoscale size of GOQDs allows them to be safely applied not only independently to cells but also to modify the surfaces of other materials. Furthermore, the simple production method, easy access, and low cost of GOQDs make them valuable for widespread application in BTE. In the present study, we further explored the differences in physical and chemical structures between Y-GOQDs and B-GOQDs and showed that Y-GOQDs promoted the osteogenic differentiation of hPDLSCs more effectively than did B-GOQDs. These findings demonstrated that Y-GOQD-treated cell sheet-laden GelMA effectively repaired periodontal bone defects in rats and further revealed the molecular biological mechanism by which Y-GOQDs promote the osteogenic differentiation of hPDLSCs by regulating mitochondrial dynamics. This study verified the possibility and effectiveness of GOQDs acting as bioactive factors in BTE both in vivo and in vitro, explored a better way to package seed cells with GOQDs, and preliminarily revealed the mechanism by which GOQDs promote hPDLSC osteogenic differentiation. The results of this study not only enrich the theoretical basis for the application of GOQDs in the field of BTE but also provide the necessary experimental basis and technical support for the treatment of periodontal bone defects in the clinic.

### Supplementary Information

**Additional file 1: Figure S1.** Phase-contrast microscopy images. hPDLSCs were cultured in osteogenic differentiation medium with different concentrations of Y-GOQDs (A) or B-GOQDs (B) for 10 days. Phase-contrast microscopy images were taken before ALP Staining. Yellow arrows represent round and contracted dead cells.

**Additional file 2: Figure S2.** Surgical procedure for modeling and hydrogel mixture implantation in the mandibular periodontal bone defects in mice.

**Additional file 3: Figure S3.** Biocompatibility evaluation in vivo.

## Data Availability

The data that support the findings of this study are available from the corresponding authors on reasonable request.

## Abbreviations

BTE: Bone tissue engineering
GOQDs: Graphene oxide quantum dots
hPDLSCs: Human periodontal ligament stem cells
GO: Graphene oxide
MSC: Mesenchymal stem cell
UV: Ultraviolet
GelMA: Gelatin methacryloyl
FBS: Fetal bovine serum
OM: Osteogenic medium
AM: Adipogenic medium
AFM: Atomic force microscopy
ECM: Extracellular matrix
XPS: X-ray photoelectron spectroscopy
ALP: Alkaline phosphatase
SEM: Scanning electron microscopy
TEM: Transmission electron microscopy
BV: Bone volume
TV: Tissue volume
GQDs: Graphene quantum dots
