# Supplementary material for: Human periodontal ligament stem cell sheets activated by graphene oxide quantum dots repair periodontal bone defects by promoting mitochondrial dynamics dependent osteogenic differentiation
Source: J Nanobiotechnology. 2024 Mar 27;22:133. doi: 10.1186/s12951-024-02422-7 (PMC10976692; doi:10.1186/s12951-024-02422-7)
Supplement: Supplementary file 1 — Additional file 1: Figure S1. Phase-contrast microscopy images. hPDLSCs were cultured in osteogenic differentiation medium with different concentrations of Y-GOQDs (A) or B-GOQDs (B) for 10 days. Phase-contrast microscopy images were taken before ALP Staining. Yellow arrows represent round and contracted dead cells. [file 12951_2024_2422_MOESM1_ESM.pdf]

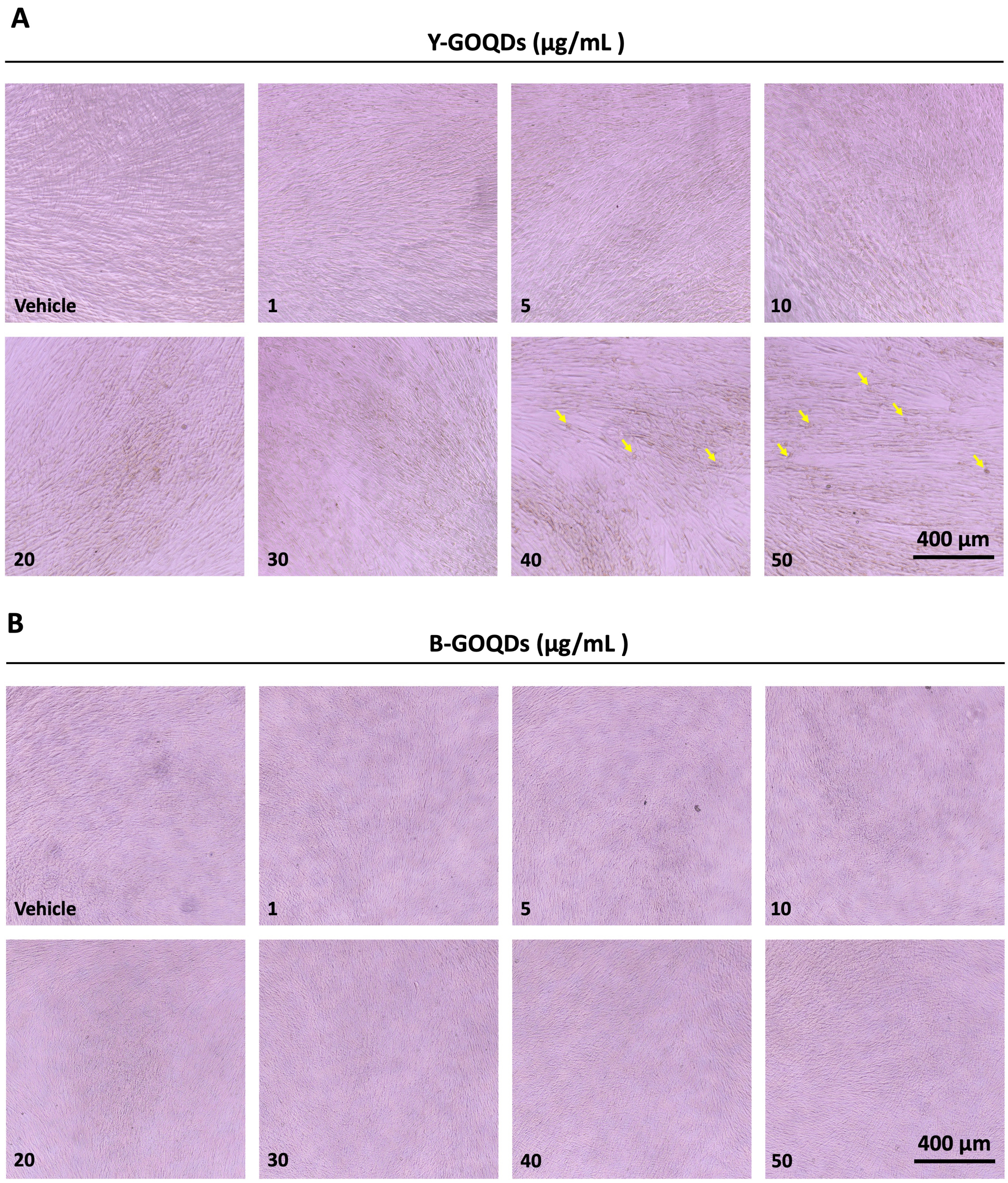

Figure S1. Phase-contrast microscopy images. hPDLSCs were cultured in osteogenic differentiation medium with different concentrations of Y-GOQDs (A) or B-GOQDs (B) for 10 days. Phase-contrast microscopy images were taken before ALP Staining. Yellow arrows represent round and contracted dead cells.
