# Supplementary material for: Human periodontal ligament stem cell sheets activated by graphene oxide quantum dots repair periodontal bone defects by promoting mitochondrial dynamics dependent osteogenic differentiation
Source: J Nanobiotechnology. 2024 Mar 27;22:133. doi: 10.1186/s12951-024-02422-7 (PMC10976692; doi:10.1186/s12951-024-02422-7)
Supplement: Supplementary file 2 — Additional file 2: Figure S2. Surgical procedure for modeling and hydrogel mixture implantation in the mandibular periodontal bone defects in mice. [file 12951_2024_2422_MOESM2_ESM.pdf]

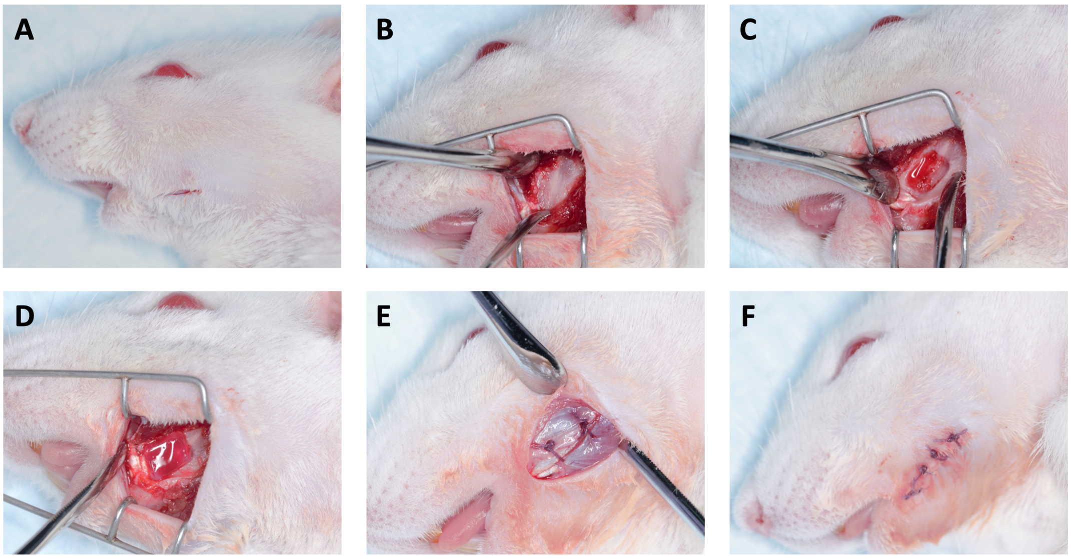

Figure S2. Surgical procedure for modeling and hydrogel mixture implantation in the mandibular periodontal bone defects in mice.

A) Skin incision. B) Separation of the muscle layers. C) High-speed turbine grinding to create a rectangular bone defect in the mandible. D) Implantation of the hydrogel mixture. E) Muscular suture. F) Skin suture.
