# Supplementary material for: Human periodontal ligament stem cell sheets activated by graphene oxide quantum dots repair periodontal bone defects by promoting mitochondrial dynamics dependent osteogenic differentiation
Source: J Nanobiotechnology. 2024 Mar 27;22:133. doi: 10.1186/s12951-024-02422-7 (PMC10976692; doi:10.1186/s12951-024-02422-7)
Supplement: Supplementary file 3 — Additional file 3: Figure S3. Biocompatibility evaluation in vivo. [file 12951_2024_2422_MOESM3_ESM.pdf]

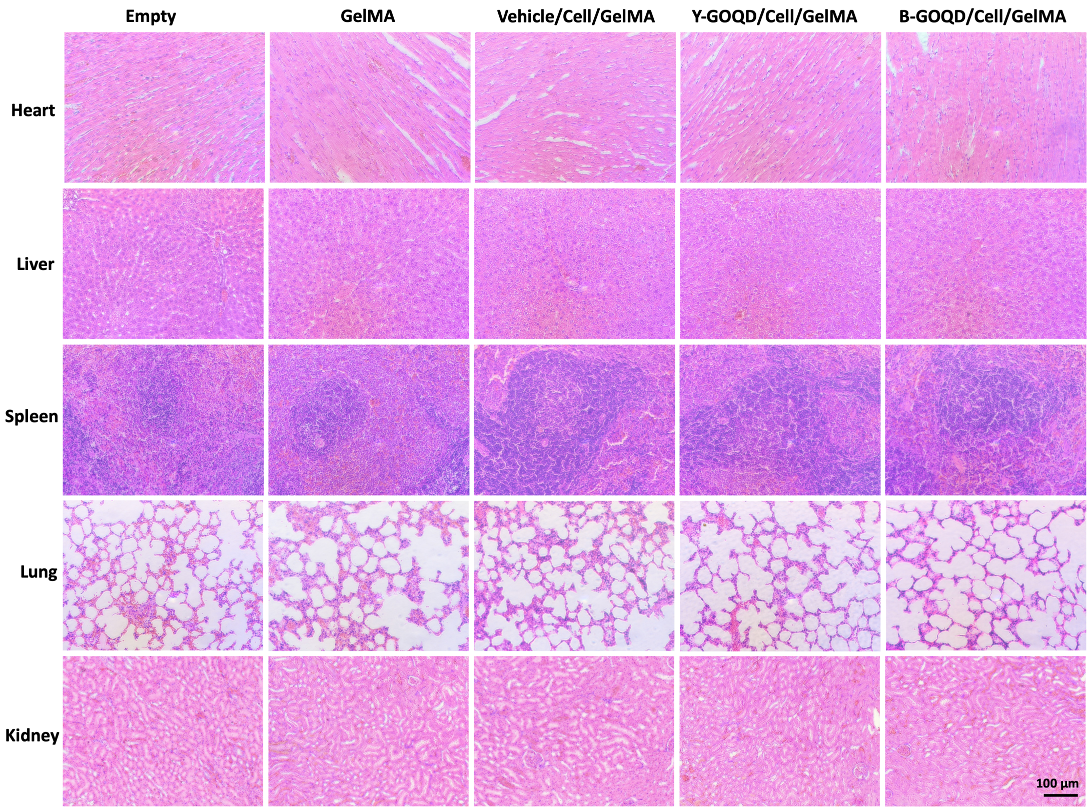

Figure S3. Biocompatibility evaluation in vivo. H&E staining of important organs of rats for 4 weeks after surgery.
